# Alcohol and the risk of sleep apnoea: a systematic review and meta-analysis

**DOI:** 10.1016/j.sleep.2017.12.005

**Published:** 2018-02

**Authors:** Evangelia Simou, John Britton, Jo Leonardi-Bee

**Affiliations:** UK Centre for Tobacco and Alcohol Studies, Division of Epidemiology and Public Health, University of Nottingham, Hucknall Road, Nottingham NG5 1PB, UK

**Keywords:** Alcohol, Sleep apnoea, Systematic review, Meta-analysis

## Abstract

**Objective:**

A systematic review and meta-analysis of the association between alcohol consumption and risk of sleep apnoea in adults.

**Methods:**

We searched Medline, EMBASE and Web of Science databases from 1985 to 2015 for comparative epidemiological studies assessing the relation between alcohol consumption and sleep apnoea. Two authors independently screened and extracted data. Random effects meta-analysis was used to estimate pooled effect sizes with 95% confidence intervals (CI). Heterogeneity was quantified using I^2^ and explored using subgroup analyses based on study exposure and outcome measures, quality, design, adjustment for confounders and geographical location. Publication bias was assessed using a funnel plot and Egger's test.

**Results:**

We identified 21 studies from which estimates of relative risk could be obtained. Meta-analysis of these estimates demonstrated that higher levels of alcohol consumption increased the risk of sleep apnoea by 25% (RR 1.25, 95%CI 1.13–1.38, I^2^ = 82%, p < 0.0001). This estimate's differences were robust in alcohol consumption and sleep apnoea definitions, study design, and quality but was greater in Low and Middle Income Country locations. We detected evidence of publication bias (p = 0.001). A further eight included studies reported average alcohol consumption in people with and without sleep apnoea. Meta-analysis revealed that mean alcohol intake was two units/week higher in those with sleep apnoea, but this difference was not statistically significant (p = 0.41).

**Conclusion:**

These findings suggest that alcohol consumption is associated with a higher risk of sleep apnoea, further supporting evidence that reducing alcohol intake is of potential therapeutic and preventive value in this condition.

## Highlights

• Alcohol consumption increased the risk of sleep apnoea by 25%.
• Findings were robust to differences in definitions of alcohol consumption and sleep apnoea.
• Association appeared stronger low and middle income countries.
• Timing and regularity of alcohol consumption likely to be important to its effect on OSA.
• Recommend advising against alcohol in people with, or at risk of, OSA.

## 1 Introduction

Obstructive sleep apnoea (OSA) is a disorder characterised by repeated episodes of partial or complete upper airway collapse or obstruction resulting in hypopnoea or apnoea during sleep [1], [2]. A diagnosis of OSA is confirmed by demonstration of apnoea's (10-s interruptions of breathing) on overnight polysomnography [3], [4], or of apnoea or hypopnoea resulting in a 3% reduction in oxygen saturation on overnight oximetry [1], - occurring five or more times per hour [1], [5]. The frequency of these events [the apnoea index (AI) or apnoea/hypopnoea index (AHI)] is also used to grade the severity of OSA: AHI ≥ 5 to <15 episodes being graded as mild, AHI ≥ 15 to <30 as moderate, and over AHI ≥ 30 as severe [6], [7].

The prevalence of OSA in the USA has been estimated at around 15% in men and 5% in women for people aged 30–70 years [7]. OSA is present in the 41% patients with a body mass index greater than 28 [3]. OSA is also more common in older aged people, current smokers, and in those with coronary artery disease, stroke, hypertension, and diabetes mellitus [4]. OSA is thought to be more common among people who consume alcohol, possibly because alcohol increases upper airway collapsibility [4], [8], [9] and also because alcohol intake can contribute to a higher body mass index. However, evidence on the effect of alcohol consumption on the risk of OSA remains mixed, with some studies reporting an increased risk in those who consume alcohol [5], [10], [11], and others finding a decreased risk [12], [13].

To clarify and quantify the association between alcohol consumption and the risk of OSA we have therefore carried out a systematic review and meta-analysis of comparative epidemiological studies including data on alcohol consumption affects and OSA in adults.

## 2 Methods

The systematic review and meta-analysis was conducted in adherence with the PRISMA [14] and MOOSE [15] guidelines. The protocol was published in the National Institute for Health Research International prospective register of systematic reviews (PROSPERO) under the registration number: 42015029910.

### 2.1 Inclusion criteria and search strategy

We included all longitudinal, cohort, case control, and cross sectional studies assessing the association between alcohol consumption and sleep apnoea in adults aged 18 years and over identified from comprehensive searches of Medline (accessed via Ovid), EMBASE (accessed via Ovid), and Web of Science databases between December 1985 and December 2015. We used search filters for observational study designs [16] and search terms for both outcome and exposure developed from relevant Cochrane Reviews groups [17]. We also searched the reference lists of included studies. No language restrictions were imposed; and foreign language papers were translated into English as necessary. The full search term strategy is presented in Supplementary Table 1. Two authors (ES and JL-B) independently screened the titles and abstracts to identify potentially relevant studies. The full text of these studies was then screened independently by the same authors. Any disagreements were resolved through discussion. Studies of populations with HIV, hepatitis B and hepatitis C viruses were excluded, as these represent selected populations, thus may not be generalizable to the general population.

### 2.2 Measures of outcome and exposure

Objective measures of OSA severity were assessed using the Respiratory Distress Index (RDI), where applicable, or Apnoea-Hypopnoea Index (AHI), in which a higher score indicated increased severity. Where possible, an AHI ≥ 5 or RDI ≥ 5 was used to define the presence of OSA, and a score <5 to define absence of OSA.

For categorical measures of alcohol consumption, where possible we defined these using the dichotomy of any alcohol versus no alcohol (reference group). However, for studies which did not report data for those who consumed no alcohol, we used the lowest exposed group as the reference group. Continuous measures of alcohol consumption were standardised, into units per week using recognised definitions: one drink being defined as 0.6 ounces, 14.0 g or 1.2 tablespoons of pure alcohol [18] or using the UK guidelines we defined one unit as eight grams of ethanol [19].

For additional analyses, categorical measures of alcohol consumption were further defined as levels of consumption: light/moderate/heavy drinking; alcohol dependence; or drinks/grams of ethanol per day, week or year. For continuous data we standardized alcohol to grams of ethanol per week. Where alcohol intake was expressed in daily terms, we multiplied these by seven to obtain a per-week figure. Heavy drinking was defined as weekly consumption of 15 or more drinks for men, and eight or more for women, whereas binge drinking either as five or more drinks during a single occasion for men, and four or more for women. Excessive drinking was defined as the presence of either binge or heavy drinking [18]. Also, according to Dietary Guidelines for Americans, moderate alcohol drinking defined as consuming less than one drink to two drinks per day for men and women respectively [20].

### 2.3 Data extraction

Data extraction was carried out in duplicate by ES and JL-B using a previously piloted form. Key data elements extracted included: study design, definition of exposure (alcohol) and outcome (OSA), geographic location, reference population, setting, number of people recruited, demographic of study population, finding, and identified limitations.

### 2.4 Quality assessment

Assessment of methodological quality was carried out using the Newcastle-Ottawa Quality Scale [21], with separate criteria for longitudinal/cohort, case control, and cross sectional studies. The maximum attainable score for longitudinal/cohort and case control studies was nine stars, and for cross sectional studies seven stars. A high quality study was deemed to be identified by a score of at least six. Methodological quality was independently assessed by ES and JL-B, with any disagreement resolved through discussion. Where the same population was used in multiple publications, we included the publication with the highest methodological quality. Due to lack of information, we did not assess the quality of studies published only in abstract forms.

### 2.5 Statistical analysis

Results were extracted from the individual studies as either adjusted measures of effect, crude measures of effect, or using raw data. Effect estimates adjusted for smoking and other factors were used in preference. Binary effect measures were extracted as odds ratios (OR), hazard ratios (HR) or risk ratios, with 95% confidence intervals (CI). Where effect measures were not presented in the paper, we estimated crude RRs for cohort and cross sectional studies and ORs for case control studies. Where exposure to alcohol was reported using quantiles or categories, we extracted adjusted effect measures relating to a comparison of the highest to the lowest exposure group. Continuous effect measures were estimated as mean differences (MD) with 95% CI. Where more than two categories of exposure to alcohol were presented within a study, we combined the categories using standard formula to estimate pooled means and the pooled standard errors [17].

Pooled measures of effect across studies were estimated using random effects meta-analysis using the generic inverse variance method to weight the studies. A random effects model was deemed appropriate due to anticipated differences in effect measures related to the inherent biases within the observational study designs included in the review. We pooled ORs and risk ratios together to estimate pooled Relative Risks (RRs) since the OSA was not assumed to be common (<5%); however HRs were not pooled with other effect measures.

Heterogeneity between the studies was quantified using I^2^ [22]. As systematic reviews combine studies that are diverse both clinically and methodologically, and it is likely that the strength of association between alcohol and OSA varies by participant characteristics; heterogeneity is anticipated [22]. Where high levels of heterogeneity were seen (I^2^>50%), subgroup analysis was used according to study quality, study design, adjustment for confounders, ascertainment of alcohol levels (excessive vs low/moderate) and OSA (objective versus subjective) and geographical location of the study population. We also carried out subgroup analyses of studies in which control groups comprised individuals who never consumed alcohol, and those in which the controls were individuals with lower alcohol intake. To assess whether the association between alcohol and OSA was independent of Body Mass Index (BMI), we performed a sensitivity analysis restricted to studies which provided BMI-adjusted measures of effect. We also performed sensitivity analyses restricted to studies which provided smoking-adjusted measures of effect. Funnel plots were used to visually assess evidence of publication bias, where at least ten studies were included in the meta-analysis. Also, Egger's statistical test was used for the assessment of publication bias. Review Manager (version 5.3) and STATA (version 14) were used to perform analyses. P values < 0.05 were taken to represent statistical significance.

## 3 Results

### 3.1 Overview of included studies

**Fig. 1:**
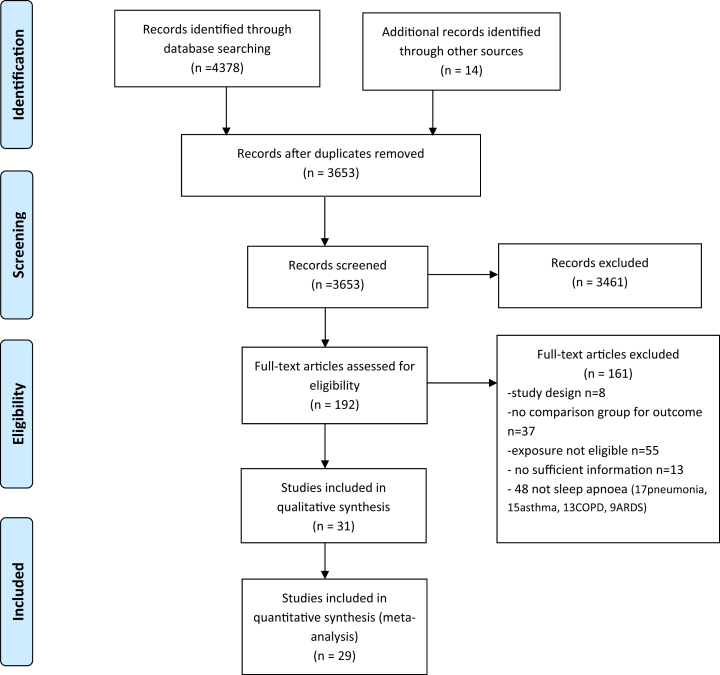
Flow chart of studies.

From an initial 4378 studies identified from the literature searches, 3639 had potentially eligible titles and abstracts, and 178 had potentially eligible full texts. After the full text screening and the exclusion of non-eligible papers, 31 studies met our criteria for inclusion in the systematic review (Fig. 1).

Twenty of the included studies used a cross sectional design [10], [11], [13], [23], [24], [25], [26], [27], [28], [29], [30], [31], [32], [33], [34], [35], [36], [37], [38], [39], nine were case controlled [5], [12], [40], [41], [42], [43], [44], [45], [46], and two were cohort studies [47], [48]. Eleven were conducted in Asia (five studies in Korea, two studies in India, one study in Israel, one study in Taiwan, one study in Japan, and one study in Pakistan) [5], [11], [24], [25], [28], [29], [30], [38], [39], [40], [42]; ten in US [13], [23], [27], [34], [35], [36], [37], [44], [47], [48], four in Australia [33], [43], [45], [46]; five in Europe (Denmark, United Kingdom, Ireland, Norway, Germany) [10], [12], [26], [32], [41], and one in Africa [31].

Most studies assessed alcohol consumption by self-report using a standardized questionnaire; one study measured alcohol dependence used International Classification of Diseases (ICD-9) codes [13]. Fifteen studies reported the quantity of alcohol as a continuous measure (number of drinks, units of alcohol or grams of ethanol consumed) and 11 studies used categories [5], [11], [13], [27], [29], [32], [34], [36], [37], [38], [40] including; current drinking [5], [34], [37]; alcohol abuse [40]; alcohol dependence [13], harmful alcohol [30], excessive [32], binge drinking [36], habitual drinking [11], [38], and regular drinking [31]. The remaining five studies did not provide additional information about how alcohol was quantified [25], [28], [29], [42], [46]. Ten of the 31 included studies reported alcohol consumption as a binary exposure, comparing any intake with no intake [5], [24], [25], [27], [28], [29], [34], [39], [42], [47]. The results presented in Table 1.

**Table 1:** Characteristics of the included studies.

Study& Year	Study design	Geographical location	Population	Alcohol ascertainment	Alcohol definition	Sleep apnoea ascertainment	Sleep apnoea definition	Adjustment
Baik, 2014 [5]	Case control	Asia/Korea	General population	Self-report	Current drinkers vs no drinkers	polysomnography	AHI ≥ 15 vs AHI < 5	NRG1 genotype, age, sex, BMI, neck circumference
Coughlin, 2004 [12]	Case control	Europe/United Kingdom	People recruited from clinic and general public	Self-report	Units/week 0–4 units vs 4–50 units	polysomnography	AHI ≥ 15 vs AHI < 5	
Enright, 2001 [23]	Cross sectional	North America/US	Elderly population	Self-report	≥25 drinks/week	Self-report questionnaire	–	Chronic bronchitis, clinic care, marital status
Fredheim, 2011 [26]	Cross sectional	Europe/Norway	Population recruited from Obesity/Centre	Self-report	Units/week	Polysomnography	AHI ≥ 5 vs AHI < 5	
Gilat, 2014 [40]	Case control	Asia/Israel	General population	Self-report	Alcohol abuse	polysomnography	–	Matched for: age and sex
Heiskel, 2002 [41]	Case control	Europe/ German	Hospital sleep laboratory population	Self-report	7glasses/week vs 8 to>21glasses/ week	Polysomnography	–	
Hussain, 2009 [24]	Cross sectional	Asia/Pakistan	General population	Self-report	Alcohol yes vs no	Self-report	–	Sex, age, BMI, collar size, shift work, nasal blockage, current smoker, family history of snoring
Hwang, 2010 [28]	Cross sectional	Asia/Taiwan	Hospital volunteer helpers	Self-report	Alcohol yes vs alcohol no	Polysomnography	AHI ≥ 5 vs AHI < 5	
Jennum, 1994 [10]	Cross sectional	Europe/Denmark	General population	Self-report	Beverage: 10grams/day of ethanol	Respiratory distress index	RDI ≥ 5 vs RDI < 5	
K.Kang, 2013 [42]	Case control	Asia/Korea	General population	Self-report	Alcohol yes vs alcohol no	Self-report (Berlin questionnaire)	(High risk vs low risk of OSA)	
K.Kang, 2014 [30]	Cross sectional	Asia/Korea	General population	Self-report	Harmful yes vs no harmful alcohol	Self-report Berlin questionnaire	High risk of OSA	Sex, age, educational level, occupation, shift work, smoking status, exercise, musculoskeletal diseases, diabetes, hypertension
Kang, 2014 [29]	Cross sectional	Asia/Korea	sleep clinic patients	Self-report	Alcohol yes vs alcohol no	Polysomnography	AHI ≥ 5 vs AHI < 5	
Kim, 2006 [25]	Cross sectional	Asia/Korea	General population (40–69 years)	Self-report	Alcohol yes vs no	Polysomnography	AHI ≥ 5 vs AHI<5	Age, sex, smoking, BMI, hypertension
Marshall, 2008 [33]	Cross sectional	Oceania/Australia	General population	Self-report	Grams/week	Respiratory distress index	RDI ≥ 5 vs RDI < 5	
McArdle, 2006 [43]	Case control	Oceania/Australia	Sleep clinic of a hospital	Self-report	Grams/week	Polysomnography	AHI ≥ 15 vs AHI < 5	Waist circumference, age
Mc Cague, 2014 [32]	Cross sectional	Europe/Ireland	hospital	Self-report	Excessive vs no excessive drinking	Polysomnography	AHI > 30 vs AHI < 5	Adjusted (not known the variables)
Ngahan, 2010 [31]	Cross sectional	Africa/Africa	General population	Self-report	Regular drinking	Self-report (Berlin questionnaire)	(High risk vs low risk of OSA)	Waist circumference, snoring
Nieto, 2000 [27]	Cross sectional	North America/US	Middle aged and elderly population	Self-report	Alcohol no vs >7drinks/week	Polysomnography	AHI ≥ 5 vs AHI < 5	
Yue, 2014 [34]	Cross sectional	North America/US	General population	Self-report	Current alcohol use(within the past 12 months) vs never	Self-report questionnaire	–	Gender, age, race, marital status, income, education, asthma, diabetes, hypertension
Peppard, 2000 [35]	Cross sectional	North America/US	General population	Self-report	Drinks/week	Polysomnography	AHI ≥ 5 vs AHI < 5	
Peppard, 2006 [47]	Cohort	North America/US	General population	Self-report	Drinks/day 0 drinks vs 3 drinks	Polysomnography	AHI ≥ 5 vs AHI < 5	Age, BMI, neck and waist circumference, current cigarette smoking antidepressant, antihypertensive, cholesterol-lowering medication
Popovici, 2013 [36]	Cross sectional	North America/US	General population	Self-report	Binge drinking	Self-report	–	Age, race/ethnicity, years of schooling, current employment and marital status, number of persons in the household, labor market income in the past year, and being born outside the U.S, smoking, diabetes, hypertension, depression, panic disorder, psychological or emotional abuse
Shamsuzzaman, 2014 [44]	Case Control	North America/US	Sleep laboratory patients	Self-report	Drinks/week	Polysomnography	AHI ≥ 5 vs AHI < 5	
Sharafkhaneh, 2005 [13]	Cross sectional	North America/US	General population	ICD 9 codes	Alcohol dependence	ICD 9 codes	–	Age, sex, and ethnicity
Sharma, 2006 [11]	Cross sectional	Asia/India	General population	Self-report	Habitual drinking (>100gr/day) yes vs no habitual	Polysomnography	AHI ≥ 5 vs AHI < 5	
Simpson, 2015 [45]	Case control	Oceania/Australia	Cases form sleep study/Controls from population	Self-report	Grams/week	Polysomnography	AHI ≥ 15 vs AHI < 15	
Udwadi, 2003 [38]	Cross sectional	Asia/India	General population	Self-report	Habitual vs no habitual drinking	Polysomnography	AHI ≥ 5 vs AHI < 5	
Wetter, 2004 [48]	Cohort	North America/US	General population	Self-report	Drinks/week	Polysomnography	AHI ≥ 5 vs AHI < 5	
Worsnop 1998 [46]	Case control	Oceania/Australia	General population	Self-report	–	polysomnography	AHI	
Yaggi, 2005 [37]	Cross sectional	North America/US	Middle aged population from sleep centre	Self-report	Current vs no current alcohol	polysomnography	AHI ≥ 5 vs AHI < 5	
Zenda, 2014 [39]	Cross sectional	Asia/Japan	General population	Self-report	Alcohol yes (150 grams/week) vs alcohol no	Polysomnography	AHI ≥ 5 vs AHI < 5	

Twenty three studies ascertained sleep apnoea using polysomnography either measuring its presence and severity using the Apnoea-Hypopnoea Index (AHI) [5], [11], [12], [25], [26], [27], [28], [29], [32], [35], [37], [38], [39], [40], [41], [43], [44], [45], [46], [47], [48] or the Respiratory distress index (RDI) [10], [33]. Seven studies relied on self-report [23], [24], [30], [31], [34], [36], [42] and in the remaining study, sleep apnoea was ascertained using ICD codes [13].

The methodological quality using the Newcastle-Ottawa Assessment Scale (Table 2) showed that quality of the included studies ranged from two to seven, with only one study deemed to be of high quality (overall score ≥ 6) [47]. Thirteen studies reported effect estimates adjusted for confounders [5], [13], [23], [24], [25], [30], [31], [32], [34], [36], [40], [43], [47]; with six adjusted for smoking [5], [24], [25], [30], [35], [47]. Lower scores were primarily due to lack of adjustment for confounding factors, instead using selective populations or a self-report assessment of alcohol exposure.

**Table 2:** Critical appraisal of included studies using Newcastle Ottawa scale.

Study, Year	Stars number		
	Selection^b^	Comparability^c^	Exposure^d^
Baik, 2014 [5]	3	1	1
Coughlin, 2004 [12]	2	0	1
Enright, 2001 [23]	2	1	2
Fredheim, 2011 [26]	1	0	2
Gilat, 2014 [40]	3	1	1
Heiskel, 2002 [41]	2	0	2
Hussain, 2009 [24]	1	1	2
Hwang, 2010 [28]	1	0	1
Jennum, 1994 [10]	2	0	2
K.Kang, 2013 [42]	3	0	2
K.Kang, 2014 [30]	1	2	2
Kang, 2014 [29]	1	0	2
Kim, 2006 [25]	1	2	1
Marshall, 2008 [33]	2	0	1
McArdle, 2006 [43]	3	1	1
Mc Cague, 2014^a^[32]	–	–	–
Ngahane, 2010 [31]	–	–	–
Nieto, 2000 [27]			
Pan, 2014 [34]	1	1	2
Peppard, 2000 [35]			
Peppard, 2006 [47]	2	2	2
Popovici, 2013 [36]			
Shamsuzzaman, 2014 [44]	3	0	1
Sharafkhaneh, 2005 [13]	2	1	2
Sharma, 2006 [11]	2	0	2
Simpson, 2015 [45]	2	0	0
Udwadi, 2003 [38]	1	0	2
Wetter, 2004 [48]	2	0	1
Worsnop 1998 [46]	3	0	1
Yaggi, 2005 [37]	1	0	2
Zenda, 2014 [39]	2	0	1

a Abstract only available-not quality assessment.

b Maximum 4 stars.

c Maximum 2 stars.

d Maximum 3 stars.

### 3.2 Meta-analysis findings

**Fig. 2:**
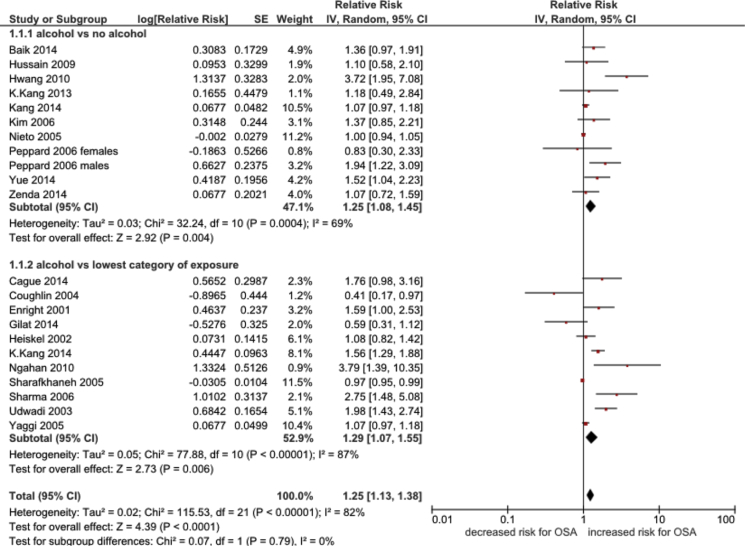
Forest plot of the association between alcohol consumption versus non-alcohol/lower alcohol consumption and the risk of OSA. *A pooled analysis of 22 comparisons from 21 studies; one study reported estimates separately for men and women [13].

A pooled analysis of the 21 studies from which relative risks could be estimated found the overall risk of OSA to be increased by 25% in people who consumed alcohol at all, or in higher amounts, than those who consumed no, or lower amounts of alcohol, respectively (pooled RR 1.25, 95% CI 1.13 to 1.38, I^2^ = 82%; Fig. 2). A stratified analysis based on the effect measure used found very similar results for studies reporting ORs (1.26, 95% CI 1.04 to 1.53, I^2^ = 79, 14 studies) and those reported risk ratios (1.27, 95% CI 1.08 to 1.49, I^2^ = 86%, seven studies).

**Fig. 3:**
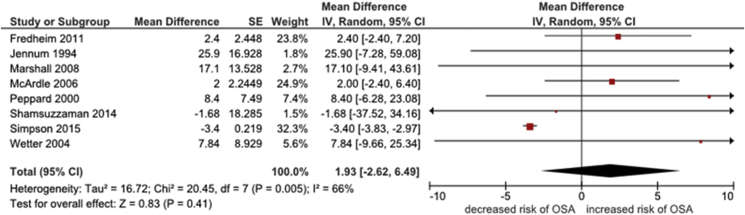
Forest plot of the association between levels of alcohol consumption in people with and without OSA.

A further eight studies presented average levels of consumption of alcohol in people with and without OSA [10], [26], [33], [35], [43], [44], [45], [48]. A meta-analysis of these studies found that people with OSA consumed, on average, approximately two units alcohol per week more than people without OSA (pooled mean difference 1.93, 95% CI -2.62 to 6.49, I^2^ = 66%, Fig. 3); however, the effect was not significant (p = 0.41). Data from the remaining two studies [36], [46] were reported in insufficient detail for inclusion in either of the above meta-analyses: one of these studies found a 9% increased risk of OSA in those who consumed alcohol compared to those with no alcohol consumption [36] while the other reported no significant association (p = 0.82) [46].

Among the 21 studies included in the pooled analysis of relative risks a sensitivity analysis showed that relative to people who did not consume any alcohol, those who consumed alcohol had 25% higher risk in OSA (pooled RR 1.25, 95%CI 1.08 to 1.45, I^2^ = 69%, 10 studies). However, in studies comparing any alcohol intake to low intake the risk of OSA was increased by 29% (pooled RR 1.29, 95%CI 1.07 to 1.55, I^2^ = 87%, 11 studies) (Fig. 2). A further sensitivity analysis restricted to the four studies which provided BMI-adjusted estimates [5], [24], [25], [47] found similar results to the main analysis, with a 41% increased risk of OSA in people who consume alcohol at all or in higher amounts (pooled RR 1.41, 95% CI 1.13 to 1.75, I^2^ = 0%, four studies). Moreover, studies which provided smoking-adjusted estimates [5], [24], [25], [47] found a marginally larger magnitude of effect compared to the main analysis (pooled RR 1.56, 95% CI 1.38 to 1.76, I^2^ = 0%, four studies).

**Fig. 4:**
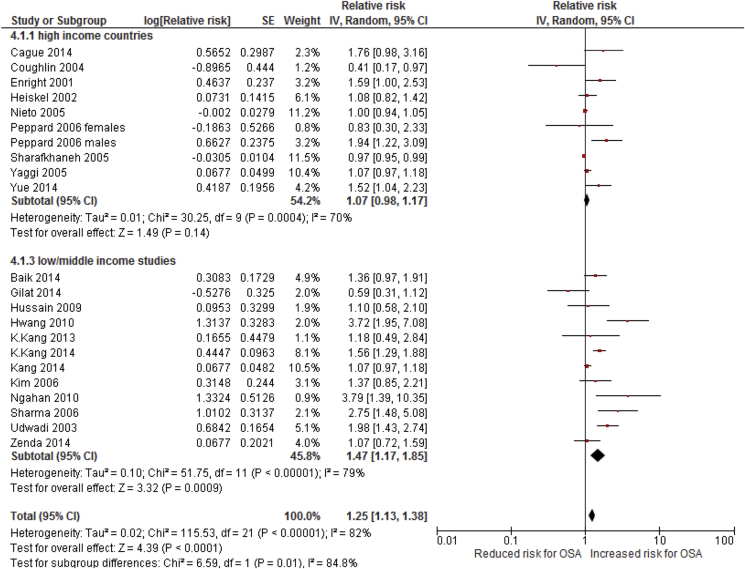
Forest plot of alcohol consumption and the risk of OSA; subgroup analysis based on high vs low/middle income countries.

Subgroup analyses of the 21 studies found that methodological quality (high versus low; p = 0.69), study design (cross-sectional, case-control, longitudinal/cohort; p = 0.25), adjustment for confounders (p = 0.40), level of alcohol consumption (excessive vs moderate/low; p = 0.65) and ascertainment of sleep apnoea (subjective vs objective; p = 0.26) did not explain high level of heterogeneity (Supplementary Table 2). Furthermore, the geographic location of the study was found to explain heterogeneity between the studies, with a quantitative interaction seen between High and Low/Middle Income Countries (p-value for subgroup differences = 0.01, Fig. 4) relating to a larger effect size in Low/Middle Income Countries (pooled RR 1.47, 95% CI: 1.17–1.85) compared to High Income Countries (pooled RR 1.07, 95% CI: 0.98–1.17). Evidence of publication bias was detected in the meta-analysis of 21 studies (Egger's test, p = 0.001).

There were insufficient data to combine study results in an exposure-response analysis, although one study estimated that for every drink consumed per day there was a 25% increased odds of at least mild sleep disordered breathing in men (p for trend = 0.006) [47]. In contrast, there was no significant exposure-response relation in women (p for trend = 0.73).

## 4 Discussion

Alcohol consumption is associated with a range of important health consequences, and a history of alcohol consumption is common among people with OSA. It is plausible that alcohol increases the risk of OSA because alcohol consumption reduces genioglossal muscle tone, predisposing patients to upper airway collapse [49] and generally increasing upper airway resistance [50]. High alcohol intakes also contribute to dietary energy intake, and hence in some cases a high body mass index, which is itself a risk factor for OSA [4]. However the extensive scientific literature of studies of the association between OSA and alcohol intake provides mixed evidence on the qualitative and quantitative nature of this association, making systematic review and meta-analysis likely to be particularly helpful in this context.

To our knowledge, this paper reports the first meta-analysis combining all available worldwide literature assessing the association between alcohol consumption and the risk of OSA, and demonstrates that people who consume alcohol, either in relation to no exposure or in those with relatively high intakes compared to those with low intakes, are approximately 25% more likely to have OSA.

Inevitably, our review findings are limited by publication bias and the low methodologic quality of the included studies, but they at least provide a comprehensive and systematic overview and synthesis of the available worldwide evidence. Another limitation of our study is the fact that only 13 of the 31 included studies adjusted for confounders; however, no appreciable difference was seen between studies which did and did not adjust for confounders. Our analysis also highlighted a high level of heterogeneity between study findings, which subgroup analysis indicated was related to economic development, with the association between alcohol and OSA appearing to be stronger in countries of low or middle income. The explanation for this finding is not clear, but confounding by obesity or other lifestyle factors is a potential factor since alcohol consumption is more likely to be limited to the most affluent in low and middle income settings. Although we expected the association between alcohol and OSA to be confounded by obesity, our restricted analysis of studies presenting BMI-adjusted estimates confirmed an independent effect of alcohol which was in fact slightly stronger than the unadjusted estimate, indicating that obesity is not the main mechanism responsible for the higher risk of OSA among those who consume alcohol.

A previous narrative review published in 2005 also found that sleep disordered breathing was likely to be more common in relation to alcohol consumption, with heavy drinkers being at particularly high risk of OSA [51]. However that study also highlighted that whilst two or three drinks before bedtime can promote sleep, this effect is not sustained after a few days of regular consumption. With more sustained regular alcohol intake, and with alcohol intake earlier in the day, alcohol consumption can be associated with insomnia [51]. It therefore appears likely that the timing and regularity of alcohol consumption are both important to the effect of alcohol on OSA, since airway muscle relaxation and reduced sensitivity to apnoea are both likely to be greatest when alcohol levels are rising, as for example after bedtime consumption [52], [53]. Our data do not enable us to estimate the nature of the exposure-response relation for either alcohol at bedtime, or alcohol in general (data being limited to single study [47]), on the occurrence or severity of OSA, however, these questions could in principle be resolved by randomised trials. We also searched for randomised controlled trials on alcohol and sleep apnoea but found none. Therefore, there is a need for the conduction of future clinical trials in order to address this important question.

Our findings thus provide confirmation that alcohol consumption may cause or exacerbate OSA, and the wider literature suggests that this may especially be the case with alcohol consumed shortly before bedtime. Whilst our findings do not confirm or refute the causality of this association, health care professionals might consider advising against bedtime alcohol among people with, or at risk of, OSA.

## Appendix A Supplementary data

mmc1
